# Analysis of the use of memes as an exponent of collective coping during COVID-19 in Puerto Rico

**DOI:** 10.1177/1329878X20966379

**Published:** 2021-02

**Authors:** José A Flecha Ortiz, Maria A Santos Corrada, Evelyn Lopez, Virgin Dones

**Affiliations:** Universidad Ana G. Mendez-Gurabo Campus, Puerto Rico

**Keywords:** classic media, collective coping, COVID-19, memes, social media

## Abstract

During the emergence of the novel COVID-19, a proliferation of memes related to events discussed in Spanish-speaking social media was observed. This study analysed the four stages of collective coping to determine how memes became triggered social representations that gradually monopolized the mainstream media. The research was performed through an electronic survey of 351 participants from Puerto Rico, which was subsequently analysed through partial least squares structural equation modeling (PLS-SEM). The study’s results reflected psychological implications in the use of memes in terms of how they were used to mitigate the stressful circumstances of a social event. This study is a pioneer in the application of Collective Coping Theory in the context of the effects that memes produce as a link to mitigate stressors. It also discusses how, when a culture faces a problem, memes become a decisive means to reinterpret the situation.

## Introduction

Memes are contagious patterns of cultural information that are transferred from mind to mind, which directly shape and transmit the key actions and mentalities of a social group (Knobel and Lankshear, 2007). With the advent of social networks, memes have become a practice to propagate and circulate communications, which has a significant dimension in cultural production and transfer (Knobel, 2006a). Knobel and Lankshear (2007) have explained that memes include things like popular melodies, key phrases, clothing fashions, ways of doing things, icons, jingles, among other elements. When the use of memes throughout social media is observed, a tendency to satirize social events using laughter as a measure of collective coping with the news of public interest becomes evident (Guilmette, 2008). Collective coping consists of the learned and uniform responses that culture manifests with the purpose of eliminating a stressor to change the interpretation of a situation. The study of collective coping is triggered by different forms of the media, which builds and communicates something (Kaplan and Liu, 2000; Lindgren, 2012; Wagner, 1998; Wagner et al., 2002).

The COVID-19 pandemic has become a stressful time for individuals, in which any media may turn into a factor that can intensify these feelings. In Puerto Rico, after the implementation of physical distancing measures, a discussion and proliferation of news was generated through the traditional and social media that prompted an increase in the dissemination of memes by audiences. These memes, which were typically shared immediately, reflected in some instances the public’s perception of a shortage of hand sanitizer and toilet paper, rapid weight gain, stressful visits to the supermarket and criticism of the government’s management of the pandemic (see Supplemental Appendix 1). Memes in social media bring a new form of communication in which digital media generate a bond between individuals and messages and a demonstration of cultural knowledge (Miltner and Highfield, 2017). According to Davison (2012), a meme becomes a replicable factor and its spread explains an observable external social phenomenon that enables actions taken by individuals, in which the transmitted idea informs their behaviour. In that sense, social networks become spaces where people participate in social construction through their experience (Tandoc & Takahashi, 2017). We argue that memes can evolve to be a measure of collective coping in response to stressful situations where humour is used to mitigate such effects. A limited number of articles have identified that the use of memes can operate in this way in response to stressful situations by turning them into spaces to share experiences, feelings and symbolic values (Ask and Abidin, 2018; Benaim, 2018; Drury, 2019). Using the Collective Coping Theory, in this study, the researchers developed a quantitative questionnaire applied to residents of Puerto Rico to: (a) identify if there was a relationship between the risk messages of COVID-19 and the use of memes as a means of collectively coping and social expression and (b) empirically validate the four stages of the Collective Coping Theory through the risk communication generated by COVID-19 and its association with the creation and observation of memes. In addition, this article discusses the background literature, as well as the results, implications and limitations of the study.

## Literature review

### Collective coping

*Coping* is defined as the thoughts and behaviours that people use to handle both internal and external stressful situations that are observed through emotional regulation and instrumental problem solving (Lazarus and Folkman, 1984). The Collective Coping Theory analyses social representations triggered by any media that build and communicate something new. According to the theory, a group tries to maintain the integrity of its vision of the world, giving meaning to any new phenomenon that challenges its established lifestyle. The study of collective coping is analysed in four stages: (a) awareness, (b) divergence, (c) convergence and (d) normalization (Wagner, 1998; Wagner et al., 2002). This study analysed the four stages of collective coping in relation to how memes were used as a collective response to reduce the stressful effects of the COVID-19 virus.

### Creation of social media and classic media awareness

Wagner et al. (2002) have defined consciousness as a phenomenon created by a claim of social relevance. At this stage, people are motivated to develop ideas and the public’s awareness is extended and amplified by different forms of the media. The traditional media (TV, radio, press), and social media and influencers have played a vital role since the appearance of COVID-19. It is noteworthy to observe that before the implementation of physical distancing measures in Puerto Rico, the United States and Europe, these media paid more attention to infections, deaths and the rapid spread of the novel virus and did not strive to create more awareness on how to avoid contagion.

Pandemic studies have identified that the role of social media and traditional media could affect perceptions of risk (Barrelet et al., 2013; Mutz, 1989; Nisbet et al., 2002) and this has been explained by the affective and cognitive effects that impact individuals (Guerrero-Rodríguez et al., 2020). Barrelet et al. (2013) has explained that risk perceptions are already influenced by the changing information collection and dissemination schemes. However, the way people perceive a risky or stressful event differs according to the medium that is used (Nisbet et al., 2002). In response, people manifest diverse cultural ways to eliminate such stresses.

Some studies on collective coping have identified that social networks become spaces of social construction (a) to facilitate participatory social action (Tandoc & Takahashi, 2017), (b) to establish knowledge about how audiences and discussion topics are organized (Lindgren, 2012) and (c) to establish collective ideas (Eriksson-Krutrök, 2020). Other research has concluded that traditional news media (a) upset the public (Wagner et al., 2002), (b) contribute to anguish (Holman et al., 2020) and (c) increase social interactions (Abdulkareem et al., 2020). These antecedents support other studies that have argued that while traditional media have a more significant effect on behaviour (Oh et al., 2015), social media have become important alternative sources of information that traditional media provide (Yoo et al., 2020). As a result, individual evaluations of a stressful event will move a person to interpretations or perceptions associated with the use of images to make sense of any new knowledge and reported facts (Caillaud et al., 2016). This medium, in turn, is central to a social participation ritual for speaking and sharing (Gasparre et al., 2010), in which people can use humour and memes, and which evolves into a form of collective coping to mitigate these effects (Benaim, 2018) and, thus, produce divergence. According to Wagner et al. (2002), divergence is the second stage of collective coping. The production of divergent images entails a grade of novelty that transcends knowledge to some degree. These variations in the grade of novelty of informed facts impact the interpretation of these messages that are metaphorically linked by a person. It has been emphasized that a meme serves as a metaphorical model (Wiggins, 2019), which explains why memes appear as a reflection of creativity and collective events through singularization. That is, a meme treats events as if they were observed for the first time, which will have an effect on a person (Rowan, 2015). These antecedents lead the researchers to propose the following:

*H1*. Exposure of the public to COVID-19 crisis messages through the traditional media, such as radio, television and press, creates awareness of risk in individuals, which impacts the observation of memes to deal with the stressful situation.
*H2*. Exposure of the public to COVID-19 crisis messages through social media creates awareness of the risks of the virus in individuals, which impacts the observation of memes to deal with the stressful situation.

### Memes and the stages of collective coping

The term *meme* appeared for the first time in the academic literature with Dawkins, (1976, 2016) who proposed *The Selfish Gene*, where he established a model of cultural change that involved the replication of ideas and knowledge. Davison (2012) has detailed that a meme has to remain for a time in the memory of an individual to be called a meme. According to its duration, it will have more power to infect other recipients of the message. On the other hand, Knobel and Lankshear (2007) have defined it as contagious patterns of cultural information that are transmitted from mind to mind and that directly shape and transmit key actions and the mentalities of a social group. Later, Miltner (2018) defined memes as mediatic objects with particular characteristics associated with ideas to notice something in a particular place. In the digital world, memes have the capability of proposing and debating an argument through visual and verbal interaction (Wiggins, 2019). Wiggins (2020) has established that to comprehend the visual composition and the discourse of the meme it is necessary to consider its semiotic composition, that is, how signs are observed, the way in which a meme is produced and communicated and how it is received by others (Blommaert, 2015). Colorado-Castellary (2010) has explained that memes are a new modality that is not limited to a global cultural network. On the contrary, they have the faculty of generating local cultural networks, as well as domestic and private ones. Wiggins (2019) has established that memes, in the same manner as a virus, have a high power of replication. The replication of memes is described as a three-stage process, which consists of assimilation (something that can be represented), expression (emerges from memory and takes a physical form, such as photo and video) and transmission (the person uses a stable physical medium to spread the message) (Davison, 2012).

Rowan (2015) has explained that memes appear as a reflection of creativity and collective events through singularization. One of their effects is that they have the power to inject humour as a collective coping mechanism to eliminate stressors (Guilmette, 2008). This happens because the use of memes creates collective identities through shared norms and values (Gal et al., 2016). Studies from the perspective of collective coping have identified that the use of memes reduces stressful effects since the use of humour allows sharing experiences, feelings and symbolic values (Ask and Abidin, 2018; Benaim, 2018; Drury, 2019). Therefore, the researchers of this study expected that through the subsequent stages (divergence, convergence and normalization), different behaviours would be observed which could provide an explanation of how society coped with stressors during the COVID-19 crisis.

#### Divergence

As noted above, Wagner et al. (2002) have defined divergence as the degree of novelty that transcends existing knowledge to a certain extent. In this stage, a group of available images is analysed to determine how they capture certain aspects of reality. The images must be impressive to attract a person (Rigutto, 2017) and generally an Internet meme is a photograph with the original image deliberately altered to inject humour (Chan, 2017). These images represent a collective coping process, and at this stage, collective emotions can be seen.

Rimé (2017) has explained that emotion usually generates a process of social exchange of emotional experiences. This is how in crises the social media become a rapid exchange of information to observe and share experiences (Huang et al., 2015). One study has identified that in collective coping processes with negative emotions and stereotypes, participants moved to a more positive emotional state (Caillaud et al., 2016). A later study concluded that the use of humour made it possible to cope with stressors in a society where COVID-19 was now perceived as more fun and less aversive (Bischetti et al., 2020). Therefore, emotions can lead to a reorganization of social knowledge, and contribute to the construction of such representations (Wagner and Kronberger, 2001), and became more predictive (Van Zomeren et al., 2008). Given that a meme can be observed as a metaphorical model with the ability to spread rapidly (Wiggins, 2019), the degree of novelty of the exposed fact and its new interpretation will be metaphorically liked to the pre-existing one, thus impacting the third stage, which is convergence (Wagner and Kronberger, 2001). It is due to this background that we further propose that

*H3*. Pre-existing knowledge about the COVID-19 crisis through the observation of memes captures aspects of a reality that will converge in the social interpretation of the majority.

#### Convergence

Wagner et al. (2002) have explained that convergence refers to the fact that some of the message’s interpretations are adopted by the majority and others are abandoned. Therefore, if a group converges on interpretation, it can take the form of interrelated metaphors, images or beliefs that converge with the social interpretation of the majority. According to Wagner et al. (2002), in the convergence stage, the resulting image or metaphor does not need to be correct or precise, but plausible. Memes create plausible collective identities through shared norms and values (Gal et al., 2016). Therefore, they stimulate comments (Bayerl and Stoynov, 2016) and amplify messages in a way that traditional media do not achieve (Leaver, 2013).

Chan (2017) has demonstrated that, according to the way in which a society faces a problem, memes can be explained by socio-psychological factors, since they help to reevaluate a stressful situation. Second, memes establish socio-political humour, since when the media oversimplify problems, society uses humour to provide a critical analysis of the facts. Finally, what Gruder et al (1978) term the ‘sleeping effect’ tracks how the persuasion of a message increases over time. This effect manifests when a message may initially be interpreted to be unbelievable or exaggerated, but is considered more accurate as time goes on (Hannah and Sternthal, 1984; Kleinnijenhuis et al., 2006; Kumkale and Albarracín, 2004).

These facts explain why people converge the use of memes to expanding available resources to mitigate stressors and then provide coping responses (Aldwin, 2007). As a result, the different interpretations of media messages, the use of memes and the decision to share them and express feelings all converge in the crisis of the COVID-19 virus. Wagner and Kronberger (2001) explain that these different interpretations tend to converge over time. The understanding of the problem can become more observable and produce normalization. Due to these antecedents, we also propose that

*H4.* The use of memes to face the stressful emotional experiences of the COVID-19 crisis loses its prevalence over time and produces normalization.

#### Normalization

Normalization explains how coping and response to emergencies occur, as these can become more scientific and less emotional (Caillaud et al., 2016). Wagner et al. (2002) have detailed that normalization reflects the response that a community perceives and that it is a challenge to the established lifestyle. Lewis et al. (2013) have established that people seek to receive validation and normalization of their experiences. The normalization process can be explained by psychological factors that each individual experiences (Pignault and Houssemand, 2017), given that a reduction in emotions leads to adaptation and the promulgation of the message, providing a feeling of control (Ashforth and Kreiner, 2002). We propose that, as time passes with the management of the COVID-19 crisis, society will take measures to face the challenge to its lifestyle and the media discourse will reduce the perception of risk, which will be less emotional, thus producing normalization.

### Model & methodology

**Figure 1.**
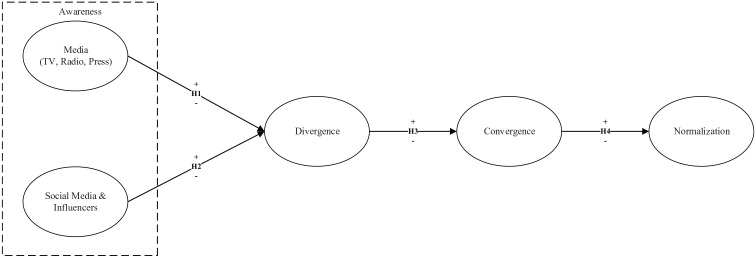
Research model *Source*: Own creation by researchers.

The model proposed in Figure 1 is guided by each of the stages proposed by Wagner et al. (2002) in order to respond to the research objectives. A total of 401 surveys were collected using a quantitative methodology by sending emails and publishing a link to the survey on social networks (Facebook, Twitter and LinkedIn). Since 50 surveys were not completed in their entirety and were therefore excluded from the sample, 351 surveys were finally used for analysis purposes. To establish a rigour in data collection, the survey was programmed and protected on the Survey Monkey platform so that it could only be accessed once. If participants left the survey or did not complete it, they were automatically ejected and could not reaccess it. This data collection lasted 7 days and was conducted 1 week after the government of Puerto Rico implemented physical distancing measures at the end of March 2020. The results were analysed through a partial least squares structural equation modeling (PLS-SEM) technique which applies a multivariant analysis that has the objective of proving research models known as structural models. The ease with which PLS-SEM is used lies in its ability to utilize reduced samples and its usefulness rests on the fact that its predictive power allows it to present more reliable results. At the end, the study’s sample was made up of participants whose ages ranged from 21 to 30 years old, *n* = 100 (28.65%); 31 to 50 years old, *n* = 52.44 (42.44%); and 51 years old or more, *n* = 67 (19.20%).

### Instrument

A total of 14 items (Table 1) were used to measure the study variables. The items were designed by the researchers based on the literature review and the project’s research objectives. All the study items were measured on a 5-point Likert-type scale, in which 1 was totally in disagreement, and 5 was totally in agreement. The construction of the instrument can be seen in Table 1, which followed the following rigour:

**Table 1.** Validity and reliability of the study.

	Coding measure	Loading factor	Cronbach’s alpha	Composite reliability	AVE
Awareness (TV radio & press)	COMR01 COMR02 COMR03 COMR04 COMR05	0.786 0.767 0.747 0.901 0.792	0.860	0.899	0.640
Awareness (social media & influencers)	COMR06 COMR07 COMR08	0.805 0.838 0.784	0.739	0.850	0.655
Divergence	TCCO03 TCCO04	0.899 0.871	0.724	0.878	0.783
Convergence	TCCO05 TCCO06	0.944 0.891	0.817	0.914	0.842
Normalization	TCCO01 TCCO02	0.879 0.841	0.649	0.85	0.739

*Source*: Own creation with SMART-PLS data.

AVE: average variance extracted.

#### Awareness

For the awareness variable, five items were used for the traditional media (TV, radio, press) and three items were used to measure social media and influencers. The definitions proposed by Wagner et al. (2002) and postulated by Gruder et al. (1978) were used for the construction of the items. The items sought to find out whether exposure to messages from the COVID-19 crisis produced stressors and if the social community perceived that the information was exaggerated, in order to observe if the public’s awareness was extended and amplified by the media.

#### Divergence

A total of two items were used to investigate if, during the COVID-19 crisis, the social group faced it with humour through the observation of memes as a way to capture aspects of reality that became a stress reliever and a form of collective coping (Chan, 2017; Wagner et al., 2002).

#### Convergence

A total of two items were used to investigate whether the social group established interpretations. The sharing of memes has been seen as a process that converges with the social interpretation of the majority, which in turn mitigates their affective factors (Wagner et al., 2002); in this case, during the COVID-19 crisis.

#### Normalization

Two items were used to determine how the response to the crisis of COVID-19 was observed and how it challenged the participants’ lifestyle, as the action became less emotional upon mitigating the stressors (Wagner et al., 2002).

### Validity and reliability

Before analysing the results, the researchers analysed the validity and reliability of the study. The summary in Table 1 shows that the alpha coefficients, the standardized weight and the convergent validity were according to the criterion of 0.70 in most of the analysis variables (Hair et al., 2016; Henseler et al., 2009). These data meant that each variable and indicator of the research model considered for the analysis maintained a high level of consistency in the results. Similarly, the average variance extracted (AVE) values reflected results over 0.50, which led the researchers to conclude that the latent variables explained more than half of the variance on their indicators, according to the criterion of 0.50 of Hair et al. (2016). The AVE values are measurements that contribute a value obtained through the questionnaire indicators. This is an ideal test given that it informs which set of indicators represent a unique underlying construction that, according to norm, should reflect values over 0.50 (Hair et al., 2016). An alpha (α = 0.64) was observed in the normalization variable. However, the data of convergent validity and AVE did not show validity problems. According to Hair et al. (2016), these measurements are better and more reliable than alpha values, which lead the researchers to conclude that validity problems were not reflected.

### Discriminant validity

The researchers found that there was no significant variance between the different variables that could have the same meaning. They executed *Heterotrait-Monotrait Ratio (HTMT) Analyzes* through PLS-SEM. The *HTMT* results were below the criterion of 0.85, consistent with the analysis of Henseler et al. (2016) and Hair et al. (2016). Therefore, there was no indication of problems between variables that could have the same meaning (Table 2).

**Table 2.** Discriminating validity.

	Convergence	Divergence	Awareness (media)	Normative	Awareness (SNS)
Convergence	–	–	–	–	–
Divergence	0.799	–	–	–	–
Awareness (media)	0.163	0.16	–	–	–
Normative	0.156	0.096	0.114	–	–
Awareness (SNS)	0.087	0.109	0.519	0.069	–

*Source*: Own creation with SMART-PLS data. SNS: Social media.

## Results

**Figure 2.**
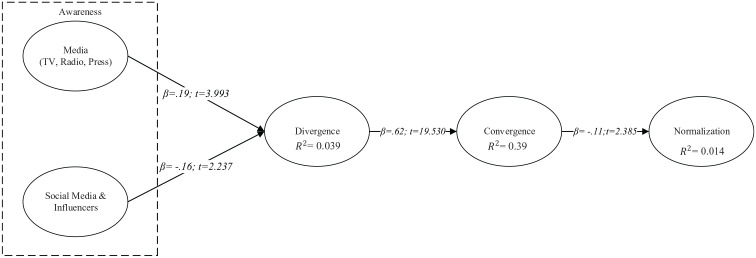
Model results. *Source*: Own creation of researchers with SMART-PLS data.

The analysis (Figure 2) began with hypotheses *H1* and *H2*, which observed whether the public’s exposure to the COVID-19 crisis messages through the traditional media, such as radio, television and the press (*H1 β* = 0.19, *p* < 0.01, *t* = 3.993, *t* > 1.960), and social media and influencers (*H2 β* = –0.16 *p* < 0.01, *t* = 2.237, *t* > 1.960) created an awareness in individuals and in turn impacted the observation of memes to cope with the stressful situation. The results support this first group of hypotheses. These data revealed an interesting aspect regarding the role of social media, suggesting that when social media and influencers (*t* = 2.237) created an awareness to cope with stress, the relationship (*β* = –0.16) to a public exposure to COVID-19 crisis messages would be weaker. These data are of interest since social media becomes an alternate means of channelling stress that traditional media do not provide. In this manner, the use of social media becomes a coping mechanism to deal with stressful situations, in which the proliferation of memes becomes a means of social expression.

The analysis then focused on how pre-existing knowledge about the COVID-19 crisis through the observation of memes captured aspects of a reality (*H3 β* = 0.62, *p* < 0.01, *t* = 19.530, *t* > 1.960) that converged with a majority of social interpretation. The third hypothesis was supported, as data revealed how the interpretation of the problem of the COVID-19 crisis transmitted a message that was adopted by the majority of users. Consequently, memes became the interpretive link of the majority, in which humour proliferated and created plausible collective identities, thus amplifying these responses to the COVID-19 crisis (Bayerl and Stoynov, 2016; Gal et al., 2016; Leaver, 2013; Wagner et al., 2002).

Finally, it was analysed whether the use of memes to face the stressful emotional experiences of the COVID-19 crisis lost its prevalence over time and became less emotional (*H4 β* = –0.11, *p* < 0.01, *t* = 2.385, *t* > 1.960) producing normalization. In this case, the hypothesis was also supported. This result is of high significance since, once normalization occurred (*t* = 2.385), the relationship of the use of memes geared towards facing the stressful emotional experiences of the COVID-19 crisis was weakened (*β* = –0.11). These data, then, confirmed the Collective Coping Theory, which states that once normalization occurs, people reduce their emotions and enter into an adaptation stage. In this stage, the message loses its prevalence and people experience a feeling of control in the face of a crisis (Ashforth and Kreiner, 2002).

## Conclusion

The study of collective coping triggered by traditional and social media provides an outline of how people manage stressors in the midst of a risk event like that generated by the COVID-19 virus. This study analysed whether there is a relationship between COVID-19 risk messages and the use of memes as a means of coping and social expression. The four stages of the Collective Coping Theory were empirically validated through risk communication generated by COVID-19 and its association with the creation and observation of memes. The results of this study provide several significant contributions to this area of communication studies.

First, the study contributes to the Collective Coping Theory by identifying a psychological implication of the effects produced by different forms of the media, such as radio, press, TV, social media and influencers in Puerto Rico, during the COVID-19 pandemic. The results reveal that since the appearance of COVID-19, people in Puerto Rico interpreted the messages of traditional media as exaggerated and failing to provide them with answers on how to effectively face the crisis. These results validated those of Chan (2017) who suggested that socio-psychological, socio-political factors and the sleeping effect can be observed through memes. It is in the initial stage of consciousness, when people take a message as unbelievable or exaggerated, that the association with the sleeping effect is seen. Therefore, over time, memes become a form of collective coping. During the distinct stages of collective coping, the socio-psychological aspects of the COVID-19 crisis are manifested through memes, as they help individuals to reevaluate a stressful situation. In the same way, memes contribute to simplifying the COVID-19 crisis, since they counteract the information that the traditional media present and provide a rational response in which humour takes relevance. This contribution matches that provided by Miltner (2018), who explained that memes prompt behaviours and certain interpretive logics that continue evolving and expanding throughout time. The proliferation of humour from memes presents social events of the government, industrial sectors and entities, thus explaining the manifestation of socio-political events.

Second, the study contributes to expanding the limited number of studies that aim to analyse collective coping and the use of humour to reduce stressful situations. Social platforms have become virtual spaces in which to share experiences, feelings and symbolic values through the proliferation of memes, even in times of crisis (Ask and Abidin, 2018; Benaim, 2018; Drury, 2019). This study is a pioneer in analysing the stages of the Collective Coping Theory within the effects produced by memes as a link to mitigate stressors. The consulted literature did not consider memes as a variable of interest in the study of collective coping. Nevertheless, the results show how, when communicating the risk of COVID-19, memes became a way to face a new and challenging phenomenon in a person’s lifestyle. The role of social media was notable, as virtual platforms became a space for social participation that stimulated comments and the participation of social construction from the experience lived by each recipient (Bayerl and Stoynov, 2016; Tandoc & Takahashi 2017). Social media amplified the message that traditional media failed to carry (Leaver, 2013).

For communication professionals and scientists, this study presents a novel vision of the effect that human behaviour produces in stressful situations triggered by media such as radio, press, TV and social media. In practice, this research reinforces how important it is for professionals to pay attention to the emotional effects of media messages and how these can trigger various behaviours in people. Communication professionals should understand that memes, as a general rule, do not generate income. Memes are born organically; they are based on a social response to an event. Therefore, this study provides a robust basis for professionals by explaining how the phenomenon occurs and identifying how society copes with the problem – with memes, a mechanism to channel and decrease stress levels and strong emotions. Memes become a new means of communication that take the form of images, video, gif, animations and photos, among other forms, in addition to emerging as a cultural phenomenon (Bommaert, 2015; Colorado-Castellary, 2010; Wiggins, 2019; 2020; Cannizzaro, 2016; Miltner, 2018).

## Limitations and future research

One of the limitations of this study is that it did not consider other variables, such as a person’s exposure to traditional and social media. This could be a variable of interest for future studies with the purpose of observing if the exposure time to different communication media in the convergence stage provides explanations about which messages are adopted and/or abandoned. Another limitation of the study is that the data were collected within a short period of time, specifically during the month of March of 2020, while social distancing and other effects of the pandemic have extended throughout a much longer period of time. Finally, the fact that the communication about the COVID-19 crisis is a novel event, limits the literary review and the applicability of previous studies.

## Supplemental Material

APENDIX – Supplemental material for Analysis of the use of memes as an exponent of collective coping during COVID-19 in Puerto Rico Click here for additional data file. Supplemental material, APENDIX for Analysis of the use of memes as an exponent of collective coping during COVID-19 in Puerto Rico by José A Flecha Ortiz, Maria A Santos Corrada, Evelyn Lopez and Virgin Dones in Media International Australia

Boostraping_Data – Analysis of the use of memes as an exponent of collective coping during COVID-19 in Puerto Rico Click here for additional data file. Boostraping_Data for Analysis of the use of memes as an exponent of collective coping during COVID-19 in Puerto Rico by José A Flecha Ortiz, Maria A Santos Corrada, Evelyn Lopez and Virgin Dones in Media International Australia https://creativecommons.org/licenses/by/4.0/ This article is distributed under the terms of the Creative
Commons Attribution 4.0 License ( http://www.creativecommons.org/licenses/by/4.0/) which
permits any use, reproduction and distribution of the work without
further permission provided the original work is attributed as specified
on the SAGE and Open Access pages ( https://us.sagepub.com/en-us/nam/open-access-at-sage).

PLS_DATA_COLC – Analysis of the use of memes as an exponent of collective coping during COVID-19 in Puerto Rico Click here for additional data file. PLS_DATA_COLC for Analysis of the use of memes as an exponent of collective coping during COVID-19 in Puerto Rico by José A Flecha Ortiz, Maria A Santos Corrada, Evelyn Lopez and Virgin Dones in Media International Australia https://creativecommons.org/licenses/by/4.0/ This article is distributed under the terms of the Creative
Commons Attribution 4.0 License ( http://www.creativecommons.org/licenses/by/4.0/) which
permits any use, reproduction and distribution of the work without
further permission provided the original work is attributed as specified
on the SAGE and Open Access pages ( https://us.sagepub.com/en-us/nam/open-access-at-sage).
