# Supplementary material for: Analysis of the use of memes as an exponent of collective coping during COVID-19 in Puerto Rico
Source: Media Int Aust. 2021 Feb;178(1):168–81. doi: 10.1177/1329878X20966379 (PMC8280555; doi:10.1177/1329878X20966379)
Supplement: APENDIX – Supplemental material for Analysis of the use of memes as an exponent of collective coping during COVID-19 in Puerto Rico [file APENDIX.pdf]

Meme 1 & 2

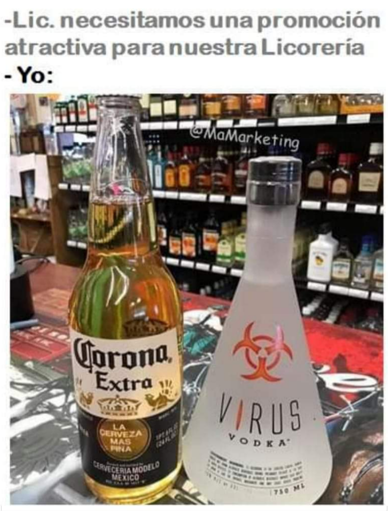

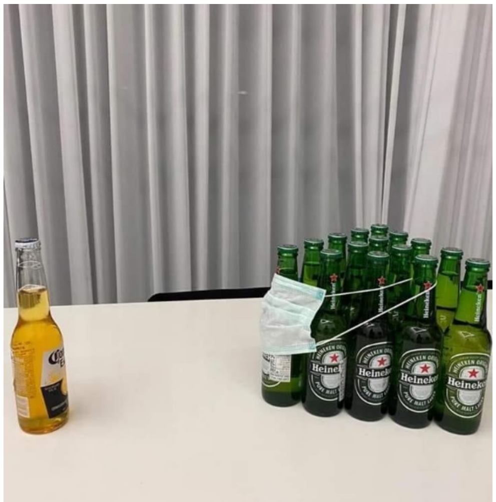

Source: Meme spread by social network WhatsApp

At the beginning of the proliferation of COVID-19 news people start by making associate with brands and the novel COVID-19

Meme 3

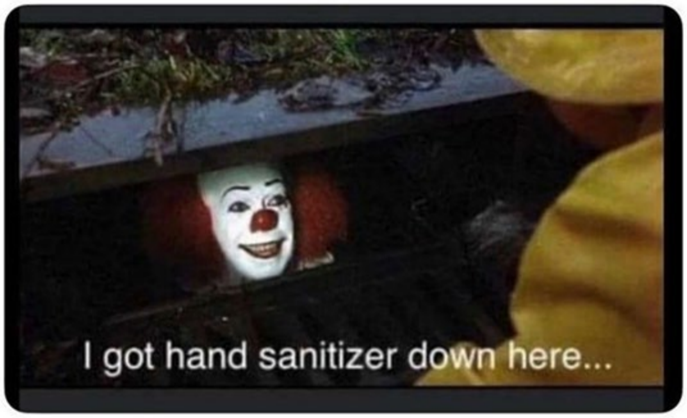

Source: Meme spread by social network WhatsApp

Very popular meme at the beginning of the pandemic complications on the theme of the scarcity of "*hand sanitizer*"

Meme 4

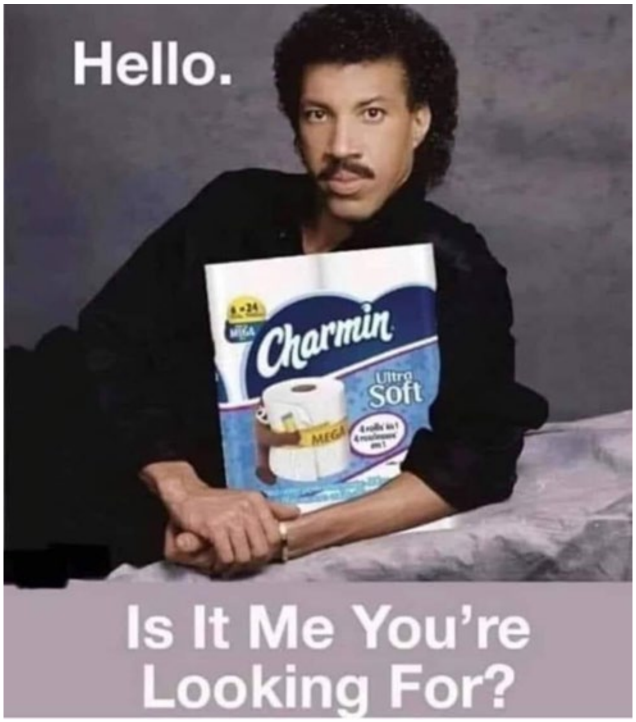

Source: Meme spread by social network WhatsApp

Memes are virialized in the face of the discussion of fictional scarcity of toilet paper

Meme 5

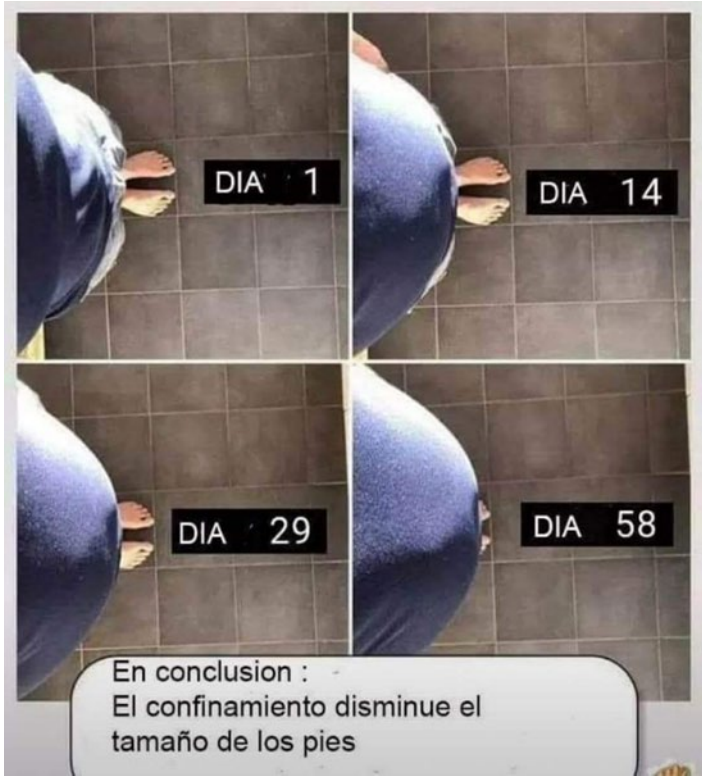

Source: Meme spread by social network WhatsApp

When the physical distance was established, people spread the problem of weight gain and lack of exercise.

Meme 6

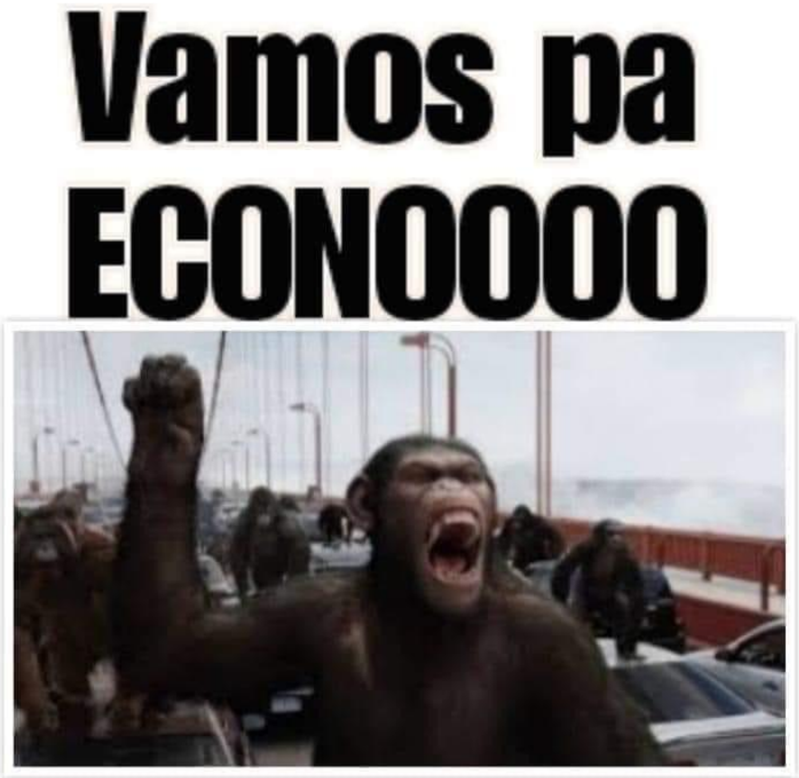

Source: Meme spread by social network WhatsApp

Econo is a network of supermarkets in Puerto Rico. They begin to spread memes *"vamos pa econo" (Go to Econo)* and other associated memes about long lines and problems to visit the supermarket and purchases without control.

Meme 7

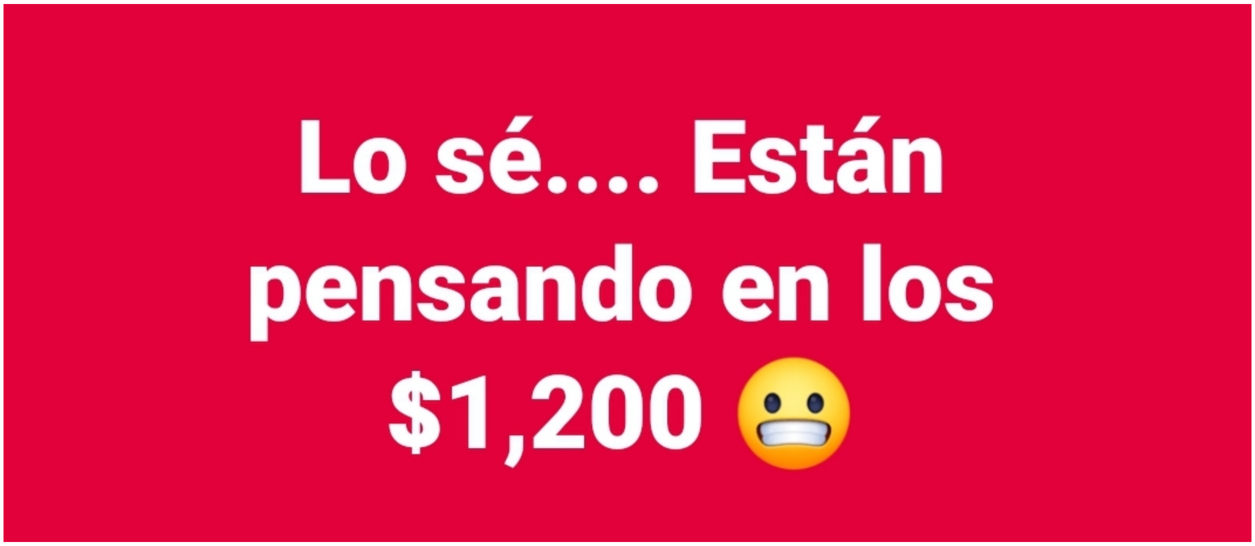

Source: Meme spread by social network WhatsApp

"Lo *se …están pensando en los 1,200*" (*I know... are thinking about the $1,200)*. The spread of memes in Puerto Rico about the announcement of the U.S. federal government stimulus in which each citizen would receive $1,200.

Meme 8

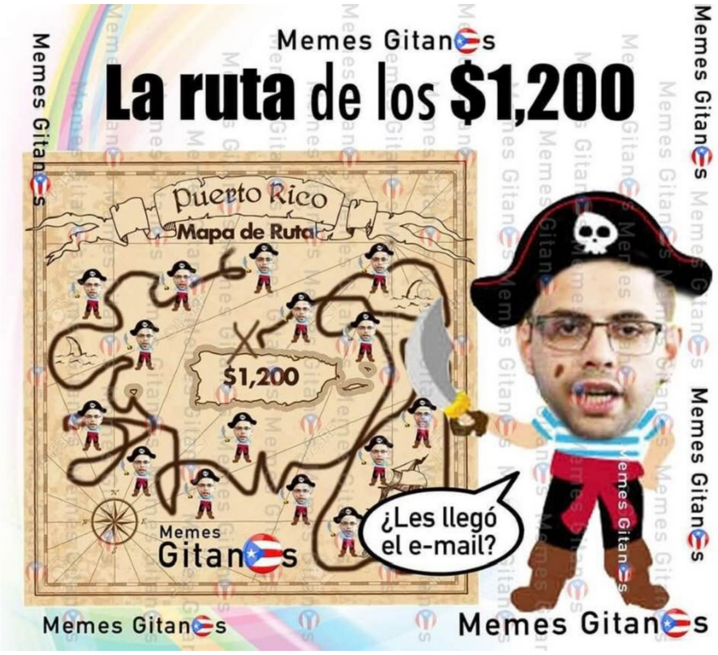

Source: Facebook Social Networkhttps://www.facebook.com/photo/?fbid=10158724759660656&set=a.10150420251495656

"*La ruta de los $ 1,200*" (the rute of 1,200) big controversy in Puerto Rico, with the secretary of the Treasury in the face of problems and lack of a plan to distribute the individual economic stimulus of $1,200

Meme 9 & 10

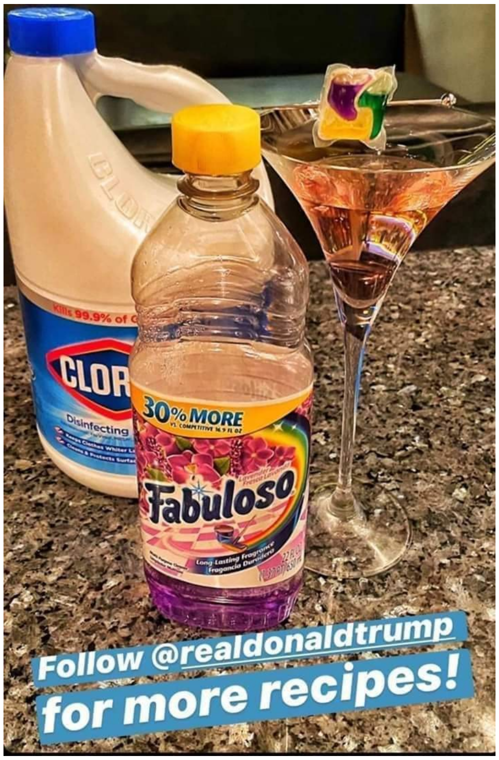

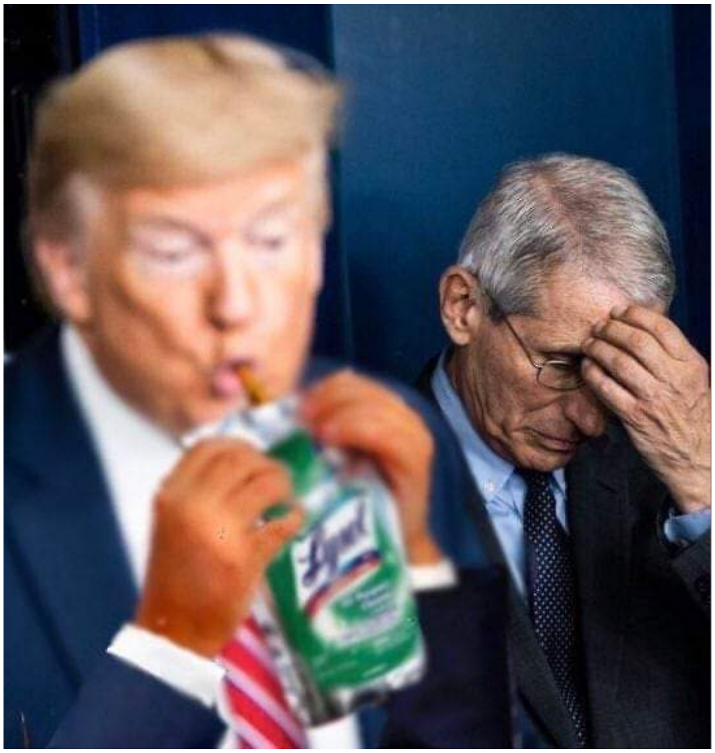

Source: Meme spread by social network WhatsApp

Meme spread on social media when President Trump at press conference recommends citizens consume disinfectants to mitigate COVID-19

Meme 11

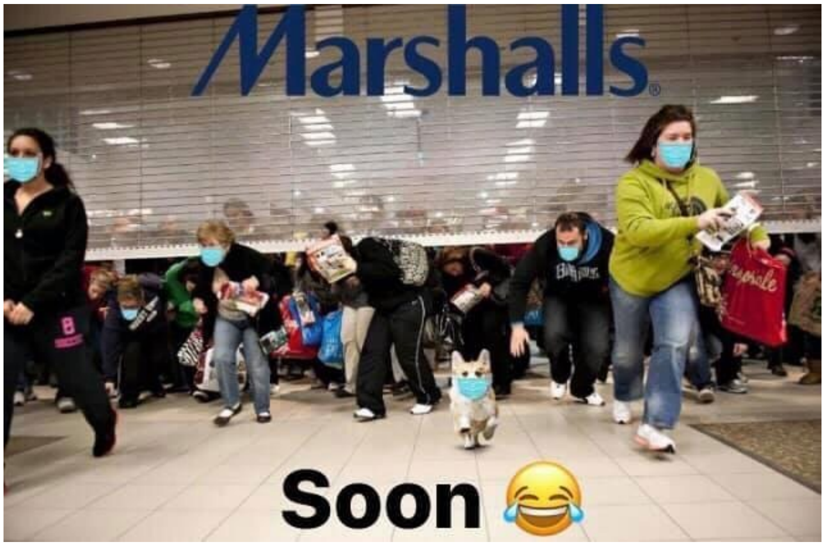

Source: Meme spread by social network WhatsApp

Meme disseminated when the government announces plan to reopen the economy in phases

Meme 12

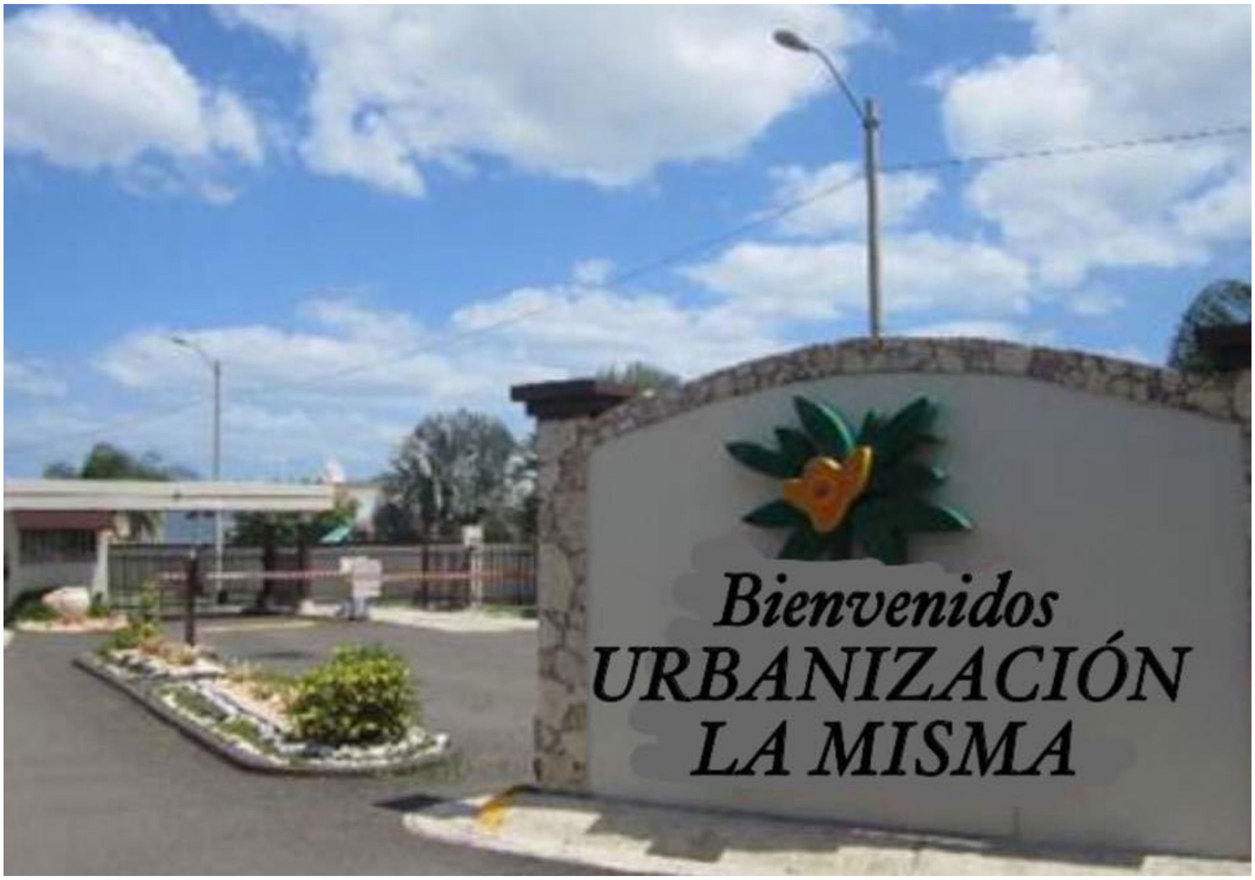

Source: Meme spread by social network WhatsApp

"La Misma" (*the same*) Many unemployed people have trouble applying for their unemployment benefits and a postal employee alerts social media amount of returned correspondence, as government employees placed between the physical and postal address "the same".
